# National surveillance data analysis of COVID-19 vaccine uptake in England by women of reproductive age

**DOI:** 10.1038/s41467-023-36125-8

**Published:** 2023-02-22

**Authors:** Laura A. Magee, Erika Molteni, Vicky Bowyer, Jeffrey N. Bone, Harriet Boulding, Asma Khalil, Hiten D. Mistry, Lucilla Poston, Sergio A. Silverio, Ingrid Wolfe, Emma L. Duncan, Peter von Dadelszen, the RESILIENT Study Group, Debra Bick, Peter von Dadelszen, Abigail Easter, Julia Fox-Rushby, Hiten D. Mistry, Eugene Nelson, Mary Newburn, Paul Seed, Marina Soley-Bori, Aricca Van Citters, Sara White

**Affiliations:** 1 Department of Women and Children’s Health, School of Life Course & Population Science, King’s College London, London, UK; 2 Biomedical Engineering Department, School of Biomedical Engineering & Imaging Sciences, King’s College London, London, UK; 3 Department of Twin Research and Genetic Epidemiology, School of Life Course & Population Science, King’s College London, London, UK; 4 Department of Obstetrics and Gynaecology, University of British Columbia, Vancouver, BC Canada; 5 The Policy Institute at King’s, Social Science and Public Policy, King’s College London, London, UK; 6 Department of Obstetrics and Gynaecology, St. George’s, University of London, London, UK; 7 Warwick Clinical Trials Unit, University of Warwick, Coventry, UK; 8 The Dartmouth Institute for Health Policy and Clinical Practice, Dartmouth, NH USA

**Keywords:** Disease prevention, Epidemiology, Vaccines, SARS-CoV-2

## Abstract

Women of reproductive age are a group of particular concern with regards to vaccine uptake, related to their unique considerations of menstruation, fertility, and pregnancy. To obtain vaccine uptake data specific to this group, we obtained vaccine surveillance data from the Office for National Statistics, linked with COVID-19 vaccination status from the National Immunisation Management Service, England, from 8 Dec 2020 to 15 Feb 2021; data from 13,128,525 such women at population-level, were clustered by age (18–29, 30–39, and 40–49 years), self-defined ethnicity (19 UK government categories), and index of multiple deprivation (IMD, geographically-defined IMD quintiles). Here we show that among women of reproductive age, older age, White ethnicity and being in the least-deprived index of multiple deprivation are each independently associated with higher vaccine uptake, for first and second doses; however, ethnicity exerts the strongest influence (and IMD the weakest). These findings should inform future vaccination public messaging and policy.

## Abstract (web summary)

Women of reproductive age may have specific concerns relating to perceived impacts on fertility and menstrual cycles that make them hesitant to receive COVID-19 vaccination. In this study, the authors explore COVID-19 vaccine uptake rates in women of reproductive age using linked data for ~13 million women in England.

## Introduction

The rapid development of effective and safe vaccines against SARS-CoV-2 greatly mitigated morbidity and mortality of the COVID-19 pandemic. From December 2020, the United Kingdom (UK) implemented an age- and vulnerability-tiered vaccination programme. Vaccination is now offered to all individuals older than five years, with a two-dose plus booster regimen^1^. By 28 August 2022, 70.1% of people aged twelve and over in England had received at least one vaccination dose, with 66.4% receiving two, and 52.2% having received three doses^2^. Consequently, and despite ongoing high SARS-CoV-2 prevalence, UK hospitalisation rates and mortality from COVID-19 have declined dramatically. Nevertheless, there were more than 11 million unvaccinated people in England alone, as of June 28, 2022^3^.

Women of reproductive age are a group of particular concern with regard to vaccine uptake.

Concerns have been expressed on social media about potentially negative impacts of COVID-19 vaccines on reproductive function (male or female), as well as menstrual irregularities. Concerns about fertility have been refuted by a substantial and reassuring body of evidence^4^. In common with other vaccinations^5^, COVID-19 vaccination may have a small impact on menstrual cycling; however, for COVID-19 vaccination, this comprised an increase in cycle length of less than one day, an increase that was within normal variation (i.e., less than eight days) for the vast majority of women, and one that was not associated with a change in menses^6^.

As only about half of pregnancies in the UK are planned^7^, regardless of pregnancy intent, it is important to acknowledge that being unvaccinated leaves women of reproductive age vulnerable to the higher risks of SARS-CoV-2 infection during pregnancy, which itself is associated with complications that include maternal mortality, severe COVID-19 illness, pre-eclampsia, preterm birth, obstetric intervention, perinatal mortality, and neonatal unit admission^8–11^. Yet, no negative effect of COVID-19 vaccination before or in pregnancy has been demonstrated, for the mother or child^12^ and vaccination is strongly recommended by the Royal College of Obstetricians and Gynaecologists (RCOG), Royal College of Midwives (RCM), and the UK Teratology Information Service (UKTIS)^13^. Pregnant women were added to the UK’s priority COVID-19 vaccination list on December 16, 2021^14^. Nevertheless, as of 3 November 2022 vaccine surveillance report in England, only 73% of women giving birth by June 2022 had received at least one vaccination, with 22% having done so prior to pregnancy. Also, only 1.0% of women unvaccinated by the time of birth had chosen to receive vaccination in the two months after pregnancy (to 26 August 2022)^2^. Elsewhere, a recent meta-analysis suggested that vaccination rates in pregnant women average only 27.5%, ranging from 7% to 68.7%^15^.

Here, we have used national vaccination data to evaluate uptake in England by women of reproductive age, to better understand barriers to vaccine acceptance in this population and inform responses to future pandemics.

## Results

We report on population-level COVID-19 vaccine uptake for 13,128,525 women of reproductive age, from 8 December 2020 (programme week 0) to 15 February 2022 (programme week 62).

Table 1 shows population demographics. Approximately one-third of women were in each of the 18–29, 30–39 and 40–49 year age groups. Two-thirds of women were of White ethnicity, with the next largest ethnic groups being Asian (~11%) and Black (4%). About 45% of women were in the lower two quintiles for IMD.

**Table 1:** Baseline characteristics of women of reproductive age eligible for COVID-19 vaccination in England, December 2020 to February 2022 (*N* (%))

	Women of reproductive age (*N* = 13,128,525)
**Age (years)**	
18–29	4,769,819 (36.3%)
30–39	4,481,214 (34.1%)
40–49	3,877,492 (29.5%)
Ethnicity^*^	
White	8,831,709 (67.3%)
British, mixed British	7,186,367 (54.7%)
Irish	69,263 (0.5%)
Any Other White origins^*^	1,576,079 (12.0%)
Black	545,229 (4.2%)
Caribbean	104,745 (0.8%)
African	324,197 (2.5%)
Any Other Black origins	116,287 (0.9%)
Asian	1,458,404 (11.1%)
Indian (Asian or Asian British)	455,635 (3.5%)
Pakistani (Asian or Asian British)	372,642 (2.8%)
Bangladeshi or British Bangladeshi	143,446 (1.1%)
Any Other Asian origins	305,653 (2.3%)
Chinese	181,028 (1.4%)
Mixed	280,844 (2.1%)
White and Black Caribbean	64,549 (0.5%)
White and Black African	47,065 (0.4%)
White and Asian	54,845 (0.4%)
Any Other mixed origins	114,385 (0.9%)
Other	385,187 (2.9%)
Any Other ethnic group	385,187 (2.9%)
Not stated/unknown	1,627,152 (12.4%)
**Index of multiple deprivation (quintile)**	
1 (most deprived)	2,901,957 (22.1%)
2	2,958,747 (22.5%)
3	2,645,594 (20.2%)
4	2,405,631 (18.3%)
5	2,216,596 (16.9%)

^*^Classified according to the Office of National Statistics, UK.

### Vaccine uptake by age

**Fig. 1:**
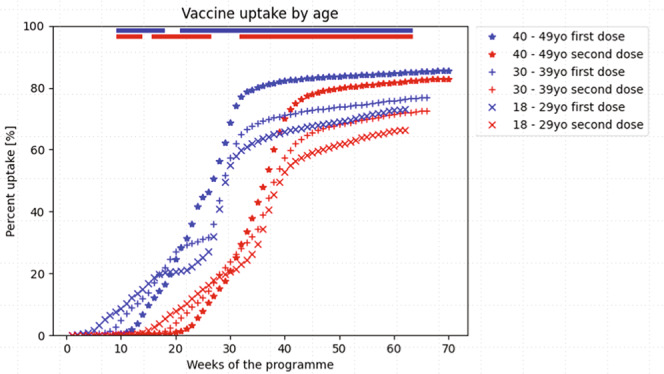
COVID-19 vaccine uptake by age, normalised for date of vaccine availability by age group (thus, week 26 represents the week for universal age-tiered vaccination offer for all women of reproductive age groups). The non-parametric Mood’s median test was used to compare the time to vaccination trajectories by age grouping; a two-sided *P* value was considered to be statistically significant was <0.001, corrected for multiple testing using the conservative Bonferroni correction (i.e., 5.38 × 10^−6^ for age); significance by week is represented by the coloured bar at the top of the Figures. The bars at the top of the figure represent the time periods during which there were significant differences in vaccine uptake by age, at the *P* < 5.38 × 10^−6^ level. Blue bars represent the first vaccination dose, and red bars second vaccination dose. It was not feasible to present 95% CI for vaccine uptake trajectories and maintain readability, but the statistical analyses accounted for trajectory variability within groups.

Vaccine uptake differed across age groups for women of reproductive age (Fig. 1). Approximately 40% of women received a first vaccination before their age-tiered eligibility date, reflecting the many women in higher-risk groups (such as frontline healthcare workers and those with underlying morbidity, as per Joint Committee on Vaccination and Immunisation (JCVI) risk categories) who were eligible for early access. Considered after date of universal availability and having adjusted for staggered age-tiered access, older women of reproductive age were more likely to be vaccinated than younger women (i.e., coverage in women aged 40–49 years of 85.5%, compared with 76.9% in women aged 30–39 years and 73% in women aged 18–29 years; *P* = 3.76 × 10^−16^ across groups and over the study period, Table 2). The pattern of vaccine uptake by age group for the second vaccination was similar; older (vs. younger) women were more likely to receive two vaccinations, with lower coverage for the second dose in younger age groups (2.6% vs. 4.3% vs. 6.7% for women aged 40–49 years, 30–39 years and 18–29 years, respectively; *P* = 1.91 × 10^−42^).

**Table 2:** Completeness of COVID-19 vaccination among women of reproductive age (18–45 years), by the week of availability in the national roll-out programme, from 8 December 2020 to 15 February 2022

Vaccine uptake	First dose	P*	Second dose	P*	Difference (1st dose minus 2nd dose uptake)	P*
**Among all women**	10,242,217 (78.0%)		9,625,999 (73.3%)		616,218 (4.7%)	
**Age** **(years)**		3.76 × 10^−16**^		1.64 × 10^−20^**		1.91 × 10^−42**^
18–29	3,482,104 (73.0%)		3,161,563 (66.3%)		320,541 (6.7%)	
30–39	3,443,878 (76.9%)		3,249,808 (72.5%)		194,070 (4.3%)	
40–49	3,316,235 (85.5%)		3,214,628 (82.9%)		101,607 (2.6%)	
**Ethnicity**		5.79 × 10^−42^**		5.77 × 10^−33^**		6.33 × 10^−14**^
White British, mixed British	6,266,542 (87.2%)		5,991,258 (83.4%)		275,284 (3.8%)	
White Irish	51,682 (74.6%)		49,052 (70.8%)		2630 (3.8%)	
Any Other White background	977,737 (62.0%)		918,210 (58.3%)		59,527 (3.8%)	
Black Caribbean	50,591 (48.3%)		43,607 (41.6%)		6984 (6.7%)	
Black African	226,267 (69.8%)		200,148 (61.7%)		26,119 (8.1%)	
Any Other Black background	70,437 (60.6%)		61,063 (52.5%)		9374 (8.1%)	
Indian (Asian or Asian British)	384,845 (84.5%)		366,261 (80.4%)		18,584 (4.1%)	
Pakistani (Asian or Asian British)	293,234 (78.7%)		262,502 (70.4%)		30,732 (8.2%)	
Bangladeshi	121,242 (84.5%)		112,074 (78.1%)		9168 (6.4%)	
Other Asian	247,804 (81.1%)		232,112 (75.9%)		15,692 (5.1%)	
Chinese	106,402 (58.8%)		94,895 (52.4%)		11,507 (6.4%)	
White and Black Caribbean	37,616 (58.3%)		33,360 (51.7%)		4256 (6.6%)	
White and Black African	33,578 (71.3%)		30,390 (64.6%)		3188 (6.8%)	
White and Asian	42,921 (78.3%)		40,388 (73.6%)		2533 (4.6%)	
Any Other Mixed background	80.036 (70.0%)		73,848 (64.6%)		6188 (5.4%)	
Any Other ethnicity	256,489 (66.6%)		236,760 (61.5%)		19,729 (5.1%)	
Not stated/unknown	994,794 (61.1%)		880,071 (54.1%)		114,723 (7.0%%)	
**Index of multiple deprivation**		3.56 × 10^−12**^		3.68 × 10^−12**^		1.9 × 10^−5^
Quintile 1 (most deprived)	2,046,834 (70.5%)		1,857,162 (64.0%)		189,672 (6.5%)	
Quintile 2	2,192,319 (74.1%)		2,035,141 (68.8%)		157,178 (5.3%)	
Quintile 3	2,093,820 (79.1%)		1,978,115 (74.8%)		115,705 (4.4%)	
Quintile 4	1,996,364 (83.0%)		1,907,340 (79.3%)		89,024 (3.7%)	
Quintile 5	1,912,880 (86.3%)		1,848,241 (83.4%)		64,639 (2.9%)	

*NS* not significant.

*The *P* value is for comparisons of vaccine uptake between subgroups of the variable examined (e.g., maternal age).

**Statistically significant results. The Bonferroni-corrected *P* values for statistical significance were 5.38 × 10^−6^ for maternal age, 1.47 × 10^−6^ for ethnicity, and 3.23 × 10^−6^ for IMD.

### Vaccine uptake by ethnicity

Vaccine uptake differed by aggregated and specific ethnic groups.

**Fig. 2:**
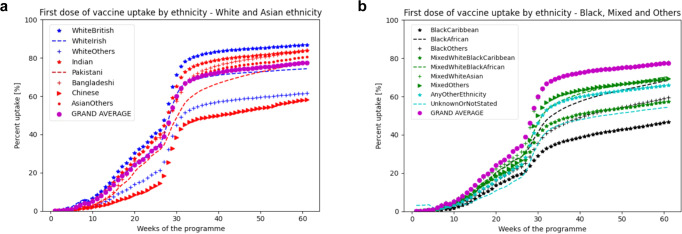
COVID-19 vaccine uptate by ethnicity, normalised for date of vaccine avalablity by age. COVID-19 first-dose vaccine uptake by ethnicity, normalised for date of vaccine availability by age group and presented for the first-dose by aggregated ethnic group (**a**) and by specific ethnic group (**b**). The “GRAND AVERAGE” represents the population average overall, presented as a reference for each graph. **a** Presents first-dose vaccine uptake by aggregated ethnic group, as in Supplementary Table 2; **b** presents first-dose vaccine uptake by specific ethnic group, as in Supplementary Table 2 with the exception of not stated/unknown which remains aggregated. The non-parametric Mood’s median test was used to compare the time to vaccination trajectories by ethnic group; a two-sided *P* value was considered to be statistically significant was <0.001, corrected for multiple testing using the conservative Bonferroni correction (i.e., 1.47 × 10^−6^ for ethnicity). Whilst it was not feasible to present 95% CI for vaccine uptake trajectories and maintain readability, the statistical analyses accounted for trajectory variability within groups. **a** The blue bars at the top of the figure represent the time periods during which there were significant differences in first-dose vaccine uptake by ethnicity, at the *P* < 1.47 × 10^−6^ level.

Considering uptake by aggregated ethnic groups, Fig. 2a shows that first-dose vaccine uptake was greatest among women of Indian background (shown by red stars), followed closely by White women (blue stars), and women of Pakistani background (red dashed line). Below the overall population average uptake (purple circles) were women of Other Asian background (red crosses), mixed ethnicity women (green stars), those of Other ethnicity (green dashes) and Black women (black stars). The lowest first-dose vaccine uptake was seen among those whose ethnicity was not stated/unknown (green crosses). Trajectories by ethnicity differed throughout the entire data collection period (*P* < 1.47 × 10^−6^), other than during a few weeks around the transition from risk-based to age-based availability for all women of reproductive age (as per the gap in the blue bar indicating statistical significance, at top of Fig. 2a).

Considering uptake by specific (non-aggregated) ethnic groups, Fig. 2b shows that there were differences in first-dose vaccine uptake by specific ethnicities not evident when considering aggregated ethnicity groups (Fig. 2a). The left panel shows vaccine uptake for White and Asian women. Vaccine uptake was greatest by White British women (blue stars), followed by women of Indian (red stars), Bangladeshi (red crosses), Other Asian (red circles), and Pakistani backgrounds (red dashes), with vaccine uptake at or above the population average (purple circles) for these women. In contrast, below-average uptake was seen by White Irish (blue dashes), White Other (blue crosses), and Chinese women (red arrows). The right panel shows vaccine uptake for Black, Mixed ethnicity, and Other ethnicity women, who also showed below-average uptake for vaccination. Mixed White and Asian women (green crosses) showed uptake similar to the population average (purple circles), with all other groups showing lower than average uptake. Uptake was similar among Mixed Other women (green arrows), Mixed White and Black African women (green dashes), and Any Other Ethnicity women (blue stars). Lower (and similar) uptake was seen by women with Black Other (black stars), Mixed White and Black Caribbean (green stars), and Not stated/unknown (blue dashes) ethnicities. Lower uptake was seen by Black Caribbean women (black stars). Patterns of uptake by ethnicity were similar for the second vaccination (Supplementary Fig. 1).

Completeness of the two-dose vaccination schedule at week 62 followed a similar pattern (Table 2), with ethnic groups least likely to have first vaccination having even lower uptake of the second dose (by 3.8–8.2%) (*P* = 6.33 × 10^−14^, Table 2).

Figure 2b also captures uptake trajectories over time, allowing inferences about time needed to reach defined vaccination levels within specific ethnicities. For example, the trajectories for Pakastani and Black African women suggest ongoing uptake, with levels likely to reach 85% within six months; in contrast, the trajectories for White Other and Chinese women appear to be plateauing, at around 60% coverage, leaving these women more vulnerable to the impact of COVID-19.

### Vaccine uptake by IMD

**Fig. 3:**
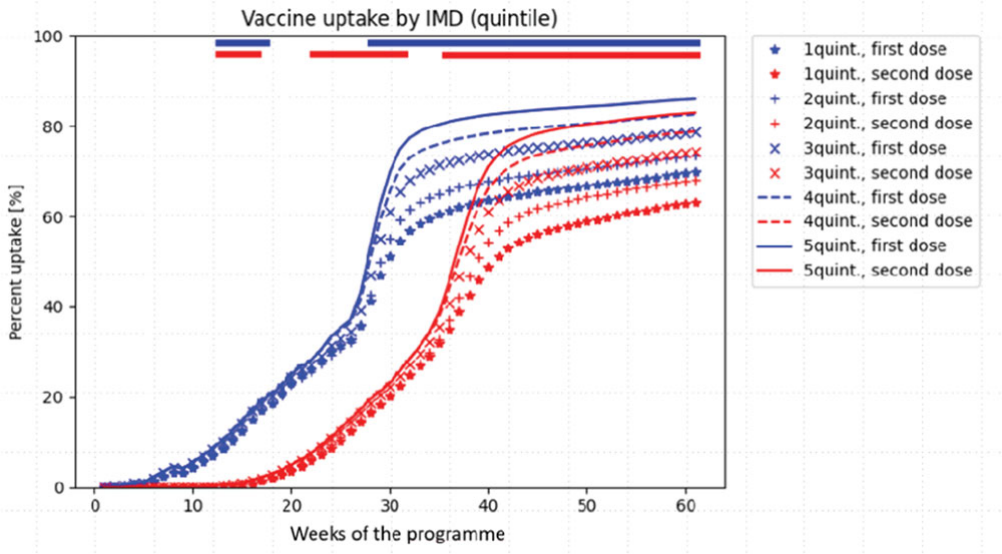
COVID-19 vaccine uptake by the index of multiple deprivation, normalised for date of vaccine availability by age group. The non-parametric Mood’s median test was used to compare the time to vaccination trajectories by IMD group; a two-sided *P* value was considered to be statistically significant was <0.001, corrected for multiple testing using the conservative Bonferroni correction (i.e., 3.23 × 10^−6^ for IMD). Whilst it was not feasible to present 95% CI for vaccine uptake trajectories and maintain readability, the statistical analyses accounted for trajectory variability within groups. The bars at the top of the figure represent time periods with significant differences in vaccine uptake by IMD, at the *P* < 3.23 × 10^−6^ level. Blue bars represent the first vaccination dose, and red bars second vaccination dose. Quintile 1 of the IMD represents the most deprived segment of the population. IMD (Index of Multiple Deprivation).

Figure 3 shows that first vaccination uptake diminished progressively, from IMD 5 to IMD 1 (*P* < 3.23 × 10^−6^), with a very similar pattern for the second vaccination. Women in IMD quintiles least likely to take up the first vaccine dose showed even lower uptake of the second dose (by 6.5% in IMD 1 to 2.9% in IMD 5), although this trend was not statistically significant (Table 2).

### Multivariate effects of age, ethnicity and IMD on vaccine untake

**Fig. 4:**
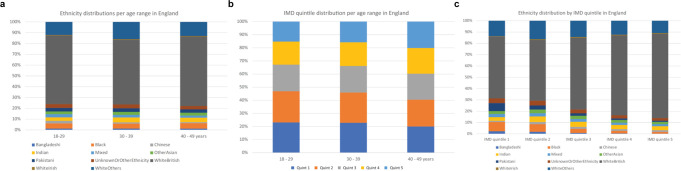
Distribution across the strata of maternal age, ethnicity and IMD by the other characteristics. **a** Distribution of ethnicity according to maternal age. **b** Distribution of IMD quintile by maternal age, with IMD 1 representing the most deprived quintile. **c** Distribution of ethnicity by IMD quintile.

Figure 4a–c demonstrates graphically that among women of reproductive age (regardless of vaccination), across strata of each of age, ethnicity, and IMD, any two of these three characteristics were not evenly distributed across the third. This was particularly true for ethnicity across IMD quintiles (Fig. 4c).

When the independent impact on any vaccination uptake (i.e., at least one dose) vs. no vaccination, of each of age, ethnicity, and IMD were considered in multivariable analyses, adjusting for vaccination programme week, any vaccine uptake of at least one dose was most likely in women aged 40–49 years and those in IMD centile 5, and least likely in minority ethnic groups, particularly women of Black Caribbean, Any Other Black, Mixed White and Black Caribbean, White Other, Chinese, Other Ethnicity, Mixed White and Black African, or Any Other Mixed origins (Table 3); for unadjusted analyses, see Supplementary Table 3. However, some specific ethnicities had a stronger effect (up to 50% reduced first-dose uptake), compared with older age (up to a 2.7-fold increased first-dose uptake) or higher IMD quintile (up to a 2.1-fold increased first-dose uptake). The model converged, but the Pearson goodness-of-fit test was significant (*P* < 0.0001).

**Table 3:** Poisson model examining the impact of maternal age, ethnicity and IMD on ANY vaccine uptake (of at least one dose)*

Counts	IRR	Robust SE	z	Two-sided *P* > | z |	95% CI	
					Lower	Upper
**Programme week**						
	1.07	0.001	119.96	<0.001	1.07	1.07
**Age band** **(ref: 18–29** **years)**						
30–39 years	1.41	0.03	17.23	<0.001	1.35	1.46
40–49 years	2.67	0.06	43.23	<0.001	2.55	2.79
**IMD 2019** **(ref: IMD 1)**						
2	1.27	0.03	9.32	<0.001	1.21	1.33
3	1.53	0.04	15.71	<0.001	1.45	1.61
4	1.78	0.05	19.96	<0.001	1.68	1.88
5	2.06	0.06	23.21	<0.001	1.94	2.19
**Ethnic origins** **(ref: White British)**						
White Irish	0.51	0.01	−32.46	<0.001	0.49	0.53
White Other	0.28	0.01	−61.27	<0.001	0.27	0.30
Black Caribbean	0.17	0.004	−77.29	<0.001	0.16	0.17
Black African	0.38	0.008	−47.84	<0.001	0.36	0.39
Any Other Black origins	0.27	0.006	−63.82	<0.001	0.26	0.28
Indian	0.79	0.02	−11.92	<0.001	0.76	0.82
Pakistani	0.59	0.01	−26.81	<0.001	0.57	0.62
Bangladeshi	0.90	0.02	−5.33	0.001	0.86	0.93
Chinese	0.25	0.006	−55.13	<0.001	0.24	0.27
Other Asian	0.65	0.01	−23.10	<0.001	0.63	0.67
White & Black Caribbean	0.28	0.006	−62.30	<0.001	0.26	0.29
White & Black African	0.41	0.008	−47.27	<0.001	0.40	0.43
White & Asian	0.58	0.007	−40.22	<0.001	0.62	0.65
Any Other Mixed origin	0.41	0.008	−46.94	<0.001	0.39	0.42
Other ethnicity	0.35	0.007	−53.46	<0.001	0.34	0.36
Not stated/unknown	0.25	0.005	−72.81	<0.001	0.25	0.26
**Constant**	0.11	0.003	−71.12	<0.001	0.11	0.12

*CI* confidence interval, *IMD* index of multiple deprivation, *IRR* incidence rate ratio, *SE* standard error.

*The model Wald chi-square was 33,063, with *P* value < 0.0001; pseudo *R*^2^: 0.8102; model convergence (9 iterations) with log pseudolikelihood = −80,037,126.

## Discussion

### Summary of findings

In this population-based study of over 13 million English women of reproductive age, univariate analyses showed COVID-19 vaccine uptake was greater in older (vs. younger) women and in the least- (vs. most-) deprived IMD quintiles. If considered by ONS aggregated ethnicity groupings, vaccine uptake was greater in White women (vs. other ethnic groups), but this obscured important differences within those aggregated groups. Amongst the aggregated group of White women, uptake was very high by White British/Mixed British women, far lower by White Irish women, and lower still by White Other women. Within the aggregated Other Asian/Asian British ethnic group, women of Bangladeshi and Indian origins had very high uptake, whereas Chinese women had uptake substantially below other Asian ethnicities. Similarly, while the aggregated group of Black women had vaccine uptake below the average overall, this was particularly low for Black Caribbean women. Although vaccination rates were continuing to improve in women of Bangladeshi, Pakistani, Black African, and Other Black origins, vaccination uptake had plateaued or was improving only slowly in most other groups by February 2022 (our study end).

Our multivariable Poisson modelling revealed that age, ethnicity, and IMD were each independently associated with any vaccine uptake, with the effect strongest for certain ethnicities and weakest for IMD. However, model goodness-of-fit testing suggested that a full explanation of the data may require additional information, such as covariates and their interactions.

Despite strong recommendations for COVID-19 vaccination of women of reproductive age, and pregnant women specifically^13, 14^, evidence of vaccination safety and efficacy outside and in pregnancy and lactation, and overwhelming evidence of the benefit of avoiding severe COVID-19 (particularly in pregnancy^8–12^), our data show that nearly a quarter of women of reproductive age in England were not vaccinated by February 2022. This was particularly evident in women under 30 years, the women who are most likely to have babies^16^. Thus, a major public health issue is to assess, understand, and encourage vaccination uptake in women of reproductive age, and to address their concerns, particularly regarding fertility and vaccine safety for maternal and infant health^17^. Also, our data highlight the importance of ongoing strategies to ensure good vaccination uptake in all ethnicity groups, requiring granular analysis of such programmes rather than aggregate approaches.

### Comparison with literature

Despite our concerns regarding a substantial population of unvaccinated women of reproductive age, the absolute rate of first vaccination coverage in women of reproductive age in our analysis (78% by February 2022) is higher than observed for the general population in England (70% by September 2022^2^). In a recent survey of pregnant women in the UK regarding attitudes towards COVID-19 vaccination for non-pregnant women of reproductive age, 81% said that they would accept or lean towards vaccination^17^; our real-world English data aligns very closely with this theoretical uptake. The same study also reported that women from ethnic minorities (compared with women from White ethnic groups) were twice as likely to reject vaccination for themselves (whether pregnant or not). Although we found significant differences in uptake between White women and women from other ethnicities, these differences were less stark.

Our univariate analyses concur with UK general population vaccination uptake data when age, ethnicity, and IMD are considered separately^2^. As each of these characteristics is unevenly distributed across the other two variables for women of reproductive age, as may be the case for the general population also; our multivariable analysis highlights the strongest contribution to vaccine uptake by some specific ethnicities, despite adjusting for age and IMD. We would also highlight that our study shows that women of reproductive age in England (2020–22) are more ethnically diverse (33% non-White background) than the general population in England and Wales (15% non-White background, 2021 census)^18^.

Differences in vaccine uptake by ethnicity are not unique to COVID-19, but have been observed with other vaccines^19^. Much effort has been directed toward encouraging COVID-19 vaccination in Black women of reproductive age^20^. Our data support the importance of this, but also reveal that women of other ethnicities may also need specific targeting. Chinese women had much lower vaccination rates than women from other Asian backgrounds, with uptake very similar to Black Caribbean women. Similarly, vaccination rates in White Other women were also extremely low. Public health campaigns focussing on individual ethnic groups, rather than a one size fits all approach, may be necessary here.

Nonetheless, our modelling confirmed that additional information beyond that collected for surveillance is required to fully explain differences in vaccine uptake. Our real-world data will have been influenced by many geopolitical issues affecting the general population, not just women of reproductive age. For example, Sinovac underwent phase 1 and phase 2 testing in an exclusively Chinese population^21^; thus, women of Chinese background may have perceived this vaccine as safest for them. In addition, women anticipating future travel may have been constrained in vaccination choice, noting not all vaccination regimens are accepted by individual governments worldwide. Information within individual communities or from external news sources (both traditional and social media) may also have influenced decision-making differentially in women of different ethnicities^22^. For example, Chinese social media prominently featured negative reports about the BNT162b2 (Pfizer) vaccine^23^; and Russian and Eastern European media prominently featured negative reports regarding both the BNT162b2 (Pfizer) and Vaxzevria (Astra Zeneca) vaccines^24^. Some Gypsy, Roma and Traveller communities in the UK (represented in White Irish or White Other ethnic groups in this study) expressed concerns about COVID-19 vaccines containing microchips and/or DNA-altering capacity^25^. Some religious groups expressed concerns ranging from vaccine manufacturing techniques (e.g., vaccines containing animal or foetal products) to vaccination appropriateness during religious fasting (e.g., during Ramadan); and positive faith-based leadership may be necessary before widespread vaccination acceptance^26^. A recent systematic review (21 studies) found reasons for poorer vaccine uptake in minority ethnic groups included concerns specific to individuals (e.g., lack of information and access), specific to the vaccines (e.g., effectiveness, safety), and specific to health systems (e.g., mistrust of formal services/government); facilitators included communication through a trusted provider, and visibility of minority ethnic groups^27^. Ongoing mixed-methods studies will help improve evidence-based vaccination decision-making and public health messaging^28^.

### Strengths and limitations

Our large and comprehensive population-based dataset: captured first and second-dose vaccination in all women of reproductive age in England; used existing ONS categories for aggregated and specific ethnicities, and for IMD classification; and enabled time-series analyses. While we did not include a time by covariate interaction, the 62-week timeframe was associated with consistent changes in age across covariates, and with little expected change in ethnicity (as a social construct) or IMD.

However, we lack data regarding other potential determinants of vaccination, including current pregnancy or breastfeeding, previous COVID-19 (personally or in close contacts), self-perceived risk of contracting COVID-19, fear of severe infection, presence of co-morbidities (including body mass index)^29^, and/or whether vaccination was offered but declined. We acknowledge that ethnicity may act as a proxy for many social determinants of health, including language and access to healthcare. We do not have data regarding the specific vaccines received; however, for most of the timeframe of this study, the BNT162b2 (Pfizer) or mRNA-1273 (Moderna) vaccines were the recommended vaccines for this age group given concerns regarding higher (though rare) thromboembolism risks with adenovirus vector vaccines in women and younger individuals^30^; Johnson & Johnson’s JNJ-78436735 single-dose mRNA vaccine only became available in the UK in late 2021.

Age, IMD and specific ethnic groupings are associated with COVID-19 vaccination in women of reproductive age in England. However, despite free and universal COVID-19 vaccine availability for months, many women remain unvaccinated. The burden of unvaccinated vulnerable women who are most susceptible to severe COVID-19 is disproportionately distributed in women from specific ethnic groupings; and a granular non-aggregated approach to vaccination may be needed to improve vaccination coverage among women of reproductive age, and by extension, at the time of birth among women who become pregnant.

Future work includes ongoing mixed-methods studies with communities to optimise informed vaccination decision-making; and again, we would caution against use of aggregated ethnic groups for this work.

## Methods

The Office for National Statistics (ONS) collects COVID-19 vaccination status (dose number and dates) from the National Immunisation Management Service (NIMS), the COVID-19 vaccination registry for England^31^. The demographic details of everyone resident in England or registered with a general practitioner (GP) in England are collected (updated daily) from the Primary Care Registration Management Service operated by NHS Digital, on behalf of NHS England. Anonymised population-level data regarding (self-identified) women of reproductive age in England and their COVID-19 vaccination status were obtained from the ONS, and measured weekly as part of national surveillance.

ONS data were grouped according to age, ethnicity, and index of multiple deprivation (IMD). Age was classified in three groups: 18–29, 30–39 and 40–49 years. Ethnicity was defined according to the set of standard codes used by the UK Government Home Office with 19 specific categories and their aggregated ethnic groups, according to NHS records (Supplementary Table 2). The IMD, also provided by ONS, uses postal code to give an overall measure of deprivation within a defined geographic area (known as a Lower-layer Super Output Area, roughly equivalent to a neighbourhood of 1000–3000 people), and incorporates the following domains: income, employment, educations skills and training, health deprivation and disability, crime, barriers to housing and services, and living environment^32^.

COVID-19 vaccination data for women of reproductive age were grouped by week, starting from the general community vaccination programme week 0 (week starting 8 December 2020); weeks were numbered sequentially thereafter. The dataset was cut on 15 February 2022. No formal sample size calculation was undertaken, noting that the dataset comprised national surveillance data from the entire population). Counts of women who received COVID-19 vaccination were considered overall and within subgroups organised by age at the start of the vaccination programme (18–29, 30–39, and 40–49 years), ethnicity (as above), and IMD (as above), with first and second vaccinations considered separately. Individual-level data were not obtained. Healthcare providers and individuals at increased risk of severe COVID-19 had access to vaccination throughout the vaccination programme, from 8 December 2020 (i.e., programme week 0). Otherwise, access was determined by age, starting with older individuals; age-tiered access to vaccination was available to women aged 40–49 years from weeks 18–19 (13–20 April 2021), to women aged 30–39 years from week 22–24 (13–26 May 2021) and to women aged 18–29 years from week 26 (8 June 2021).

Descriptive plots were created of vaccine uptake by age, ethnicity, and IMD grouping, over time. Ethnicity was evaluated in both aggregated groups and in specific categories (Supplementary Table 2), with the exception of Not stated/unknown (to simplify presentation, and because targeted public messaging for these women is not possible). As is standard practice, IMD was divided into five quintiles, with IMD 1 representing the most deprived communities. Graphs showing vaccine uptake trajectories (i.e., pattern over time) were aligned with respect to the dates of the age-tiered vaccine availability; data for women aged 40–49 years were shifted eight weeks later, and for 30–39 years, four weeks later, so that week 26 in vaccine uptake plots over time represents the time of vaccine availability for all women of reproductive age (Fig. 1).

### Analyses

The non-parametric Mood’s median test was used to compare the time to vaccination trajectories by the grouping of the parameter considered, by age, ethnicity, or IMD; a two-sided *P* value considered to be statistically significant was <0.001, corrected for multiple testing using the conservative Bonferroni correction (i.e., 5.38 × 10^−6^ for age, 1.47 × 10^−6^ for ethnicity, and 3.23 × 10^−6^ for IMD); significance by week is represented by the coloured bar at the top of the Figures. Whilst it was not feasible to present 95% CI for vaccine uptake trajectories in the Figures and maintain readability, the statistical analyses accounted for trajectory variability within groups. Vaccine uptake, for first and second doses, was calculated, overall and by age, ethnicity and IMD. Also, vaccine coverage at week 62, for first and second doses, was compared by Mood’s test. As above, *P* < 0.001 was corrected for multiple testing.

Adjusted effects on vaccine uptake were evaluated by multivariable Poisson models, applied to the counts of events within each strata. To study effects on any (vs. no) vaccine uptake, a model was designed, incorporating age (18–29, 30–39 and 40–49 years), ethnicity (specific category as in Supplementary Table 2), and IMD quintiles as covariates. The effect size was evaluated using incidence rate ratios (IRR) and 95% confidence intervals. Model convergence was checked, and satisfied over nine iterations. Goodness-of-fit testing was undertaken to examine whether there were other influences on vaccine uptake for which we lacked information. Analyses were conducted using STATA, version 17 and Python 3.8^33^.

### Ethics

Ethics approval was not required for this analysis, given the use of aggregate, anonymised data already approved and in use for surveillance of vaccine uptake. There are no exclusions to data flowing to ONS (including data from individuals who have chosen to opt out nationally) under the Statistics and Registration Service Act 2007.

### Public and patient involvement

Public and Patient Involvement was through our paid, study-specific group, beginning with study design and extending to feedback on the findings.

### Reporting summary

Further information on research design is available in the Nature Portfolio Reporting Summary linked to this article.

### Supplementary information

Supplementary Information

Reporting Summary

## Data Availability

The raw vaccine uptake data are protected and are not available due to data privacy laws. The processed (clustered, anonymised) data are available from the UK Health Security Agency to whom a research request can be made (RandD.OFFICE@ukhsa.gov.uk).

## Code availability

All code used to analyse the dataset is openly available at https://zenodo.org/record/7470642#.Y6OKEi-l3fY.

## References

[1] Coronavirus (COVID-19) vaccine. National Health Service, UK. https://www.nhs.uk/conditions/coronavirus-covid-19/coronavirus-vaccination/coronavirus-vaccine/ (2022).

[2] COVID-19 vaccine surveillance report—week 35. UK Health Security Agency https://assets.publishing.service.gov.uk/government/uploads/system/uploads/attachment_data/file/1101870/vaccine-surveillance-report-week-35.pdf (2022).

[3] Research and analysis. Population and household estimates, England and Wales: Census 2021. https://www.gov.uk/government/publications/census-2021-first-results-england-and-wales/population-and-household-estimates-england-and-wales-census-2021 (2022).

[4] BFS & ARCS Covid-19 Vaccines & Fertility—updated FAQs. https://www.britishfertilitysociety.org.uk/2021/07/27/bfs-arcs-covid-19-vaccines-fertility-2/ (2021).

[5] Suzuki S, Hosono A (2018). No association between HPV vaccine and reported post-vaccination symptoms in Japanese young women: Results of the Nagoya study. Papillomavirus Res..

[6] Edelman A (2022). Association between menstrual cycle length and covid-19 vaccination: global, retrospective cohort study of prospectively collected data. BMJ Med..

[7] Wellings K (2013). The prevalence of unplanned pregnancy and associated factors in Britain: findings from the third National Survey of Sexual Attitudes and Lifestyles (Natsal-3). Lancet.

[8] Khalil A, Blakeway H, Samara A, O’Brien P (2022). COVID-19 and stillbirth: direct vs indirect effect of the pandemic. Ultrasound Obstet. Gynecol..

[9] Perez-Lopez FR (2022). Obstetric and perinatal outcomes of pregnancies with COVID 19: a systematic review and meta-analysis. J. Matern Fetal Neonatal Med..

[10] Birol Ilter P (2022). Maternal and perinatal outcomes of SARS-CoV-2 infection in unvaccinated pregnancies during Delta and Omicron waves. Ultrasound Obstet. Gynecol..

[11] Gurol-Urganci I (2022). Obstetric interventions and pregnancy outcomes during the COVID-19 pandemic in England: a nationwide cohort study. PLoS Med..

[12] Prasad S (2022). Systematic review and meta-analysis of the effectiveness and perinatal outcomes of COVID-19 vaccination in pregnancy. Nat. Commun..

[13] COVID-19 merged information sheet and decision aid. https://www.rcog.org.uk/media/13xkcdsa/2021-02-24-combined-info-sheet-and-decision-aid.pdf (2022).

[14] UK Health Security Agency. https://www.gov.uk/government/news/pregnant-women-urged-to-come-forward-for-covid-19-vaccination (2021).

[15] Galanis P (2022). Uptake of COVID-19 vaccines among pregnant women: a systematic review and meta-analysis. Vaccines.

[16] Average age of mothers at childbirth in the United Kingdom from 1938 to 2020. *Statista* (https://www.statista.com/statistics/294590/average-age-of-mothers-uk/) (2021).

[17] Skirrow H (2022). Women’s views on accepting COVID-19 vaccination during and after pregnancy, and for their babies: a multi-methods study in the UK. BMC Pregnancy Childbirth.

[18] Population estimates by ethnic group and religion, England and Wales: 2019. Office for National Statistics. https://www.ons.gov.uk/peoplepopulationandcommunity/populationandmigration/populationestimates/articles/populationestimatesbyethnicgroupandreligionenglandandwales/2019#:~:text=As%20part%20of%20the%20White,points%20to%20an%20estimated%205.8%25 (2021).

[19] Watkinson RE, Williams R, Gillibrand S, Sanders C, Sutton M (2022). Ethnic inequalities in COVID-19 vaccine uptake and comparison to seasonal influenza vaccine uptake in Greater Manchester, UK: a cohort study. PLoS Med..

[20] COVID-19 vaccine hesitancy—debunking the myths using a community engagement approach underpinned by NICE guidance. National Institute for Health and Care Excellence. https://www.nice.org.uk/sharedlearning/covid-19-vaccine-hesitancy-debunking-the-myths-using-a-community-engagement-approach-underpinned-by-nice-guidance (2021).

[21] WHO. Background document on the inactivated vaccine Sinovac-CoronaVac against COVID-19: background document to the WHO Interim recommendations for use of the inactivated COVID-19 vaccine, CoronaVac, developed by Sinovac. https://apps.who.int/iris/handle/10665/341455 (2021).

[22] Wilson SL, Wiysonge C (2020). Social media and vaccine hesitancy. BMJ Glob. Health.

[23] Chinese media criticise Pfizer COVID-19 vaccine, tout local shots. *Reuters* https://www.reuters.com/business/healthcare-pharmaceuticals/chinese-media-criticise-pfizer-covid-19-vaccine-tout-local-shots-2021-01-20/ (2021).

[24] Facebook bans Russian firm behind mysterious Pfizer and AstraZeneca vaccine smear campaign. *itvNEWS* https://www.itv.com/news/2021-08-11/facebook-bans-russian-firm-behind-covid-vaccine-smear-campaign (2021).

[25] Gypsies, Roma, Travellers and Showmen unite to Give COVID the Jab. YouTube https://www.youtube.com/watch?v=D4-S7xdnsec (2021).

[26] Muravsky, N. L., Betesh, G. M. & McCoy, R. G. Religious doctrine and attitudes toward vaccination in Jewish law. *J. Relig. Health*10.1007/s10943-021-01447-8 (2021). 10.1007/s10943-021-01447-8 PMC8549591 34708328

[27] Kamal A, Hodson A, Pearce JM (2021). A rapid systematic review of factors influencing COVID-19 vaccination uptake in minority ethnic groups in the UK. Vaccines.

[28] COVID-19 vaccine uptake in UK’s minority ethnic groups. UK Research and Innovation https://www.ukri.org/news-and-events/tackling-the-impact-of-covid-19/vaccines-and-treatments/covid-19-vaccine-uptake-in-uks-minority-ethnic-groups/ (2021).

[29] Aw J, Seng JJB, Seah SSY, Low LL (2021). COVID-19 vaccine hesitancy—a scoping review of literature in high-income countries. Vaccines.

[30] Rizk JG (2021). Clinical characteristics and pharmacological management of COVID-19 vaccine-induced immune thrombotic thrombocytopenia with cerebral venous sinus thrombosis: a review. JAMA Cardiol..

[31] COVID-19 vaccinations. NHS England. https://www.england.nhs.uk/statistics/statistical-work-areas/covid-19-vaccinations/ (2022).

[32] The English Indices of Deprivation 2019 (IoD2019) Ministries of Housing, Communities and Local Government. https://assets.publishing.service.gov.uk/government/uploads/system/uploads/attachment_data/file/835115/IoD2019_Statistical_Release.pdf (2019).

[33] Molteni, E. Code for vaccination women childbearing age England (0.0). *Zenodo*10.5281/zenodo.7470642 (2022).

