# Author Correction: National surveillance data analysis of COVID-19 vaccine uptake in England by women of reproductive age

**DOI:** 10.1038/s41467-023-41366-8

**Published:** 2023-09-13

**Authors:** Laura A. Magee, Erika Molteni, Vicky Bowyer, Jeffrey N. Bone, Harriet Boulding, Asma Khalil, Hiten D. Mistry, Lucilla Poston, Sergio A. Silverio, Ingrid Wolfe, Emma L. Duncan, Peter von Dadelszen, the RESILIENT Study Group, Debra Bick, Peter von Dadelszen, Abigail Easter, Julia Fox-Rushby, Hiten D. Mistry, Eugene Nelson, Mary Newburn, Paul Seed, Marina Soley-Bori, Aricca Van Citters, Sara White

**Affiliations:** 1 Department of Women and Children’s Health, School of Life Course & Population Science, King’s College London, London, UK; 2 Biomedical Engineering Department, School of Biomedical Engineering & Imaging Sciences, King’s College London, London, UK; 3 Department of Twin Research and Genetic Epidemiology, School of Life Course & Population Science, King’s College London, London, UK; 4 Department of Obstetrics and Gynaecology, University of British Columbia, Vancouver, BC Canada; 5 The Policy Institute at King’s, Social Science and Public Policy, King’s College London, London, UK; 6 Department of Obstetrics and Gynaecology, St. George’s, University of London, London, UK; 7 Warwick Clinical Trials Unit, University of Warwick, Coventry, UK; 8 The Dartmouth Institute for Health Policy and Clinical Practice, Dartmouth, NH USA

**Keywords:** Disease prevention, Epidemiology, Vaccines, SARS-CoV-2

Correction to: *Nature Communications* 10.1038/s41467-023-36125-8 published online 22 February 2023

The original version of this Article omitted a statement in the Acknowledgements recognising the UK Health Security Agency for providing data. This has been added to the Acknowledgements in both the PDF and HTML versions of the Article.

