# Supplementary material for: National surveillance data analysis of COVID-19 vaccine uptake in England by women of reproductive age
Source: Nat Commun. 2023 Feb 22;14:956. doi: 10.1038/s41467-023-36125-8 (PMC9947170; doi:10.1038/s41467-023-36125-8)

SUPPLEMENTARY APPENDIX

COVID-19 vaccine uptake in England by women of reproductive age: Analysis of national surveillance data

Laura A. Magee[1]*, Professor of Women's Health, King's College London, UK (laura.a.magee@kcl.ac.uk);

Erika Molteni[2]*, Skills Development Fellow, King's College London;

Vicky Bowyer[3], Senior Clinical Research Manager – COVID Symptom Study Biobank, King's College London;

Jeff Bone[4], Biostatistical Lead at British Columbia Children's Hospital Research Institute, University of British Columbia, Canada;

Harriet Boulding[5], Senior Research Fellow, The Policy Institute at King's, UK;

Asma Khalil[6], Professor of Obstetrics and Maternal-Fetal Medicine, St. George's Hospital, University of London, UK;

Hiten D. Mistry[1], Senior Research Fellow, King's College London, UK;

Lucilla Poston[1], Professor of Maternal and Fetal Health, King's College London, UK;

Sergio A. Silverio[1], Research Associate in Social Science of Women's Health, King's College London, UK;

Ingrid Wolfe[1], Clinical Reader in Paediatrics and Child Health, King's College London, UK;

Emma L. Duncan[3]ɫ, Professor of Clinical Endocrinology, King's College London;

Peter von Dadelszen[1]ɫ, Professor of Global Women's Health, King's College London; and

the RESILIENT Study Group[#].

* These authors contributed equally.

ɫ These authors jointly supervised this work.

[#] A list of authors and their affiliations appears at the end of the paper. Collaborators are listed in Supplementary Table 1.

Affiliations:

1. Department of Women and Children's Health, School of Life Course & Population Science, King's College London, London, UK;
2. Biomedical Engineering Department, School of Biomedical Engineering & Imaging Sciences, King's College London, London, UK;
3. Department of Twin Research and Genetic Epidemiology, School of Life Course & Population Science, King's College London, London, UK;
4. Department of Obstetrics and Gynaecology, University of British Columbia, Canada;
5. The Policy Institute at King's, Social Science and Public Policy, King's College London, London, UK;
6. Department of Obstetrics and Gynaecology, St. George's University of London, London, UK.

Address for correspondence:
Professor Laura A. Magee
Addison House

Guy's Campus, Great Maze Pond
London, UK
SE1 1UL

Table of contents

	**TITLE**	**PAGE NUMBER**
**Supplementary Tables**		
Table 1	RESILIENT Study Group collaborators	3
Table 2	Office for National Statistics classificaiton of self-declared ethnicity	4
Table 3	Poisson model (unadjusted) examining the impact of vaccine programme week, age, ethnicity, and IMD on ANY vaccine uptake (of at least one dose)	5
**Supplementary Figures**		
Figure 1	COVID-19 second dose vaccine uptake by ethnicity	6

Supplementary Table 1: RESILIENT Study Group Collaborators

Collaborator	Position
Mr. Colin NJ Campbell	UK Health Security Agency, COVID Vaccine Coverage Team, Public Health England, UK
Ms. Helen Campbell	UK Health Security Agency, COVID Vaccine Coverage Team, Public Health England, UK
Dr James MN Duffy	Specialist Registrar, Obstetrics and Gynaecology, North Middlesex Hospital, London, UK
Professor Glyn Elwyn	Co-director of the Coproduction Laboratory within The Dartmouth Institute for Health Policy and Clinical Practice, Hanover, USA
Mr. Jamie LopezBernal	UK Health Security Agency, COVID Vaccine Coverage Team, Public Health England, UK
Dr Nathalie MacDermott	Academic Clinical Lecturer, KCL
Mr Ken Hodson	Head of UK Teratology Information Service, UK
Professor Rebecca Reynolds	Personal Chair of Metabolic Medicine, The University of Edinburgh, UK
Professor Claire J Steves	Consultant Physician and Endocrinologist, Co-Lead, COVID Symptom Study Biobank, KCL
Ms. Rachel Roberts	Research Manager, Tommy's Charity, UK

UK (United Kingdom)

Supplementary Table 2: Office for National Statistics classification of self-declared ethnicity

**Broad ethnic category**	**Ethnicity group**	
White	A	British, Mixed British
	B	Irish
	C	Any other White background*
Black/African/Caribbean/Black British	M	Caribbean
	N	African
	P	Any other Black background
Indian (Asian or Asian British)	H	Indian or British Indian
Pakistani (Asian or Asian British)	J	Pakistani or British Pakistani
Other Asian/Asian British	K	Bangladeshi or British Bangladeshi
	L	Any other Asian background
	R	Chinese
Mixed/multiple ethnic groups	D	White and Black Caribbean
	E	White and Black African
	F	White and Asian
	G	Any other mixed background
Any other ethnic group	S	Any other ethnic group
Not stated/unknown	T	NULL (Unknown)
	W	NULL (Unknown)
	Z	Not Stated

Supplementary Table 3: Poisson model (unadjusted) examining the impact of age, ethnicity, and IMD on ANY vaccine uptake*

**Counts**	**IRR**	**Robust SE**	**z**	**P>|z|**	**[95% CI]**	
					**Lower**	**Upper**
**Programme week**						
	1.03	0.000	81.15	<0.001	1.029	1.031
**Age band** (Ref: 18-29 yrs)						
30-39 yrs	1.16	0.01	13.16	<0.001	1.14	1.19
40-49 yrs	1.40	0.02	26.46	<0.001	1.37	1.44
**IMD 2019** (Ref: IMD 1)						
2	1.06	0.01	4.35	<0.001	1.03	1.09
3	1.12	0.02	7.44	<0.001	1.09	1.15
4	1.16	0.02	9.10	<0.001	1.12	1.20
5	1.19	0.02	10.14	<0.001	1.15	1.23
**Ethnic origins** (Ref: White British)						
White Irish	0.85	0.01	-14.07	<0.001	0.84	0.87
White Other	0.67	0.01	-35.19	<0.001	0.066	0.69
Black Caribbean	0.65	0.01	-39.36	<0.001	0.64	0.66
Black African	0.77	0.01	-24.28	<0.001	0.75	0.79
Any other Black origins	0.87	0.01	-12.36	<0.001	0.86	0.89
Indian	0.77	0.01	-24.29	<0.001	0.75	0.79
Pakistani	0.95	0.01	-4.63	<0.001	0.93	0.97
Bangladeshi	0.85	0.02	-13.36	<0.001	0.83	0.87
Chinese	0.96	0.01	-3.23	0.001	0.940.24	0.98
Other Asian	0.90	0.01	-9.76	<0.001	0.88	0.92
White & Black Caribbean	0.50	0.01	-58.13	<0.001	0.49	0.52
White & Black African	0.74	0.01	-25.85	<0.001	0.72	0.75
White & Asian	0.64	0.01	-40.22	<0.001	0.62	0.65
Any Other Mixed origin	0.60	0.01	-37.22	<0.001	0.59	0.62
Other ethnicity	0.72	0.01	-29.14	<0.001	0.71	0.74
Not stated/Unknown	0.63	0.07	-42.33	<0.001	0.62	0.64
**Constant**	0.16	0.03	-87.60	<0.001	0.15	0.17

CI (confidence interval), IMD (index of multiple deprivation), IRR (incidence rate ratio), SE (standard error)

** The model Wald chi-square was 16,816, with p value <0.0001; Pseudo R^2: 0.6759; model convergence (9 iterations) with log pseudolikelihood = -32,133,761.*

Supplementary Figure 1: COVID-19 <u>second dose</u> vaccine uptake by ethnicity, normalised for date of vaccine availability by age group and presented for the second dose by aggregated ethnic group (Supplementary Figure 1a) and by specific ethnic group (Supplementary Figure 1b).

<u>Supplementary Figure 1 legend</u>: The bars at the top of the figures represent time periods with significant differences in vaccine uptake by ethnicity, at the p<1.47x10^{-6} level.

Supplementary Figure 1(a) presents <u>second dose</u> vaccine uptake by aggregated ethnic group, as in Supplementary Table 1.

Supplementary Figure 1(b) presents <u>second dose</u> vaccine uptake by specific ethnic group, as in Supplementary Table 1 with the exception of 'Not stated/Unknown' which remains aggregated.

(a)

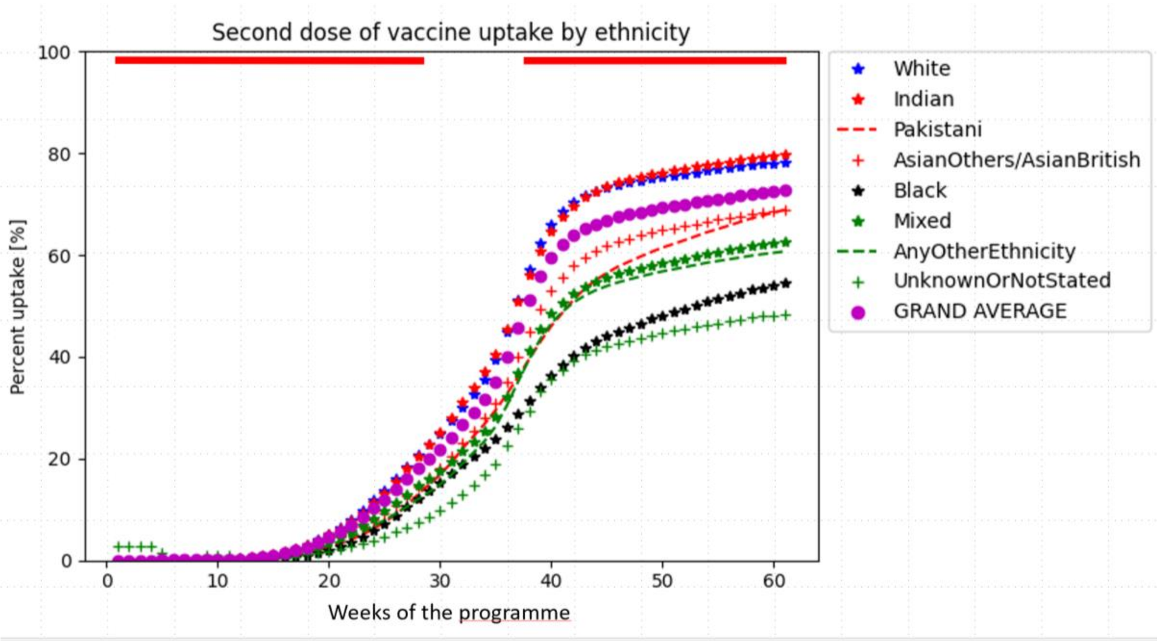

(b)

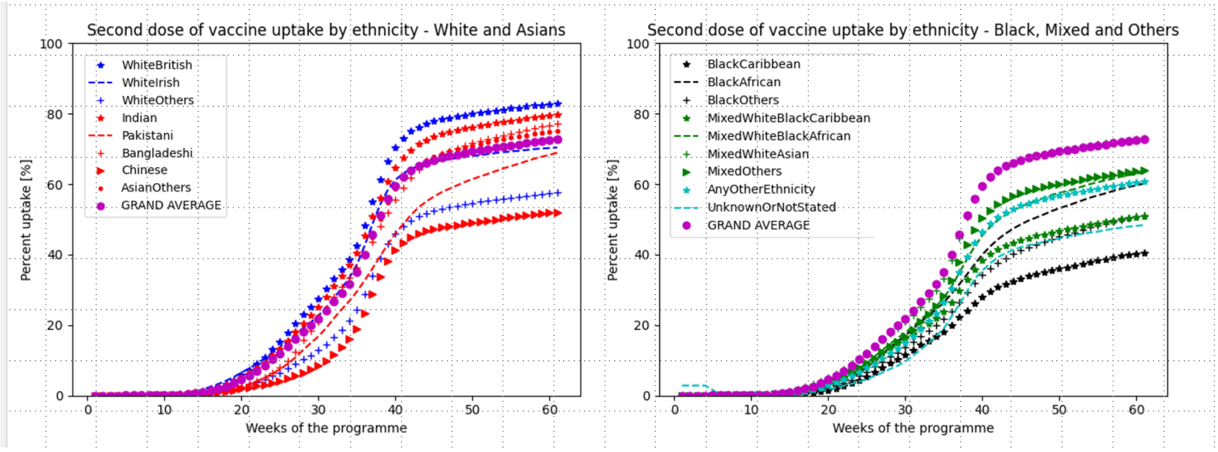

Supplement: Supplementary file 1 — Supplementary Information [file 41467_2023_36125_MOESM1_ESM.pdf]
