# Supplementary material for: National surveillance data analysis of COVID-19 vaccine uptake in England by women of reproductive age
Source: Nat Commun. 2023 Feb 22;14:956. doi: 10.1038/s41467-023-36125-8 (PMC9947170; doi:10.1038/s41467-023-36125-8)
Supplement: Supplementary file 2 — Reporting Summary [file 41467_2023_36125_MOESM2_ESM.pdf]

nature portfolio

Corresponding author(s): Laura A. Magee, NCOMMS-22-43154A

Last updated by author(s): Dec 18, 2022

Reporting Summary

Nature Portfolio wishes to improve the reproducibility of the work that we publish. This form provides structure for consistency and transparency in reporting. For further information on Nature Portfolio policies, see our Editorial Policies and the Editorial Policy Checklist.

Statistics

For all statistical analyses, confirm that the following items are present in the figure legend, table legend, main text, or Methods section.

n/a	Confirmed	
☐	☒	The exact sample size (*n*) for each experimental group/condition, given as a discrete number and unit of measurement
☐	☒	A statement on whether measurements were taken from distinct samples or whether the same sample was measured repeatedly
☐	☒	The statistical test(s) used AND whether they are one- or two-sided<br>*Only common tests should be described solely by name; describe more complex techniques in the Methods section.*
☐	☒	A description of all covariates tested
☐	☒	A description of any assumptions or corrections, such as tests of normality and adjustment for multiple comparisons
☐	☒	A full description of the statistical parameters including central tendency (e.g. means) or other basic estimates (e.g. regression coefficient) AND variation (e.g. standard deviation) or associated estimates of uncertainty (e.g. confidence intervals)
☐	☒	For null hypothesis testing, the test statistic (e.g. *F*, *t*, *r*) with confidence intervals, effect sizes, degrees of freedom and *P* value noted<br>*Give P values as exact values whenever suitable.*
☒	☐	For Bayesian analysis, information on the choice of priors and Markov chain Monte Carlo settings
☒	☐	For hierarchical and complex designs, identification of the appropriate level for tests and full reporting of outcomes
☒	☐	Estimates of effect sizes (e.g. Cohen's *d*, Pearson's *r*), indicating how they were calculated

Our web collection on statistics for biologists contains articles on many of the points above.

Software and code

Policy information about availability of computer code

Data collection	The Office for National Statistics (ONS) collects COVID-19 vaccination status (dose number and dates) from the National Immunisation Management Service (NIMS), the COVID-19 vaccination registry for England.
Data analysis	Analyses were conducted using STATA, version 17 and Python 3.8[33]. All code used to analyse the dataset is openly available at https://zenodo.org/record/7470642#.Y6OKEi-l3fY.

For manuscripts utilizing custom algorithms or software that are central to the research but not yet described in published literature, software must be made available to editors and reviewers. We strongly encourage code deposition in a community repository (e.g. GitHub). See the Nature Portfolio guidelines for submitting code & software for further information.

Data

Policy information about availability of data

All manuscripts must include a data availability statement. This statement should provide the following information, where applicable:

- Accession codes, unique identifiers, or web links for publicly available datasets
- A description of any restrictions on data availability
- For clinical datasets or third party data, please ensure that the statement adheres to our policy

The raw vaccine uptake data are protected and are not available due to data privacy laws. The processed (clustered, anonymised) data are available at https://

research.UKHSA.gov.uk/contact/.

Human research participants

Policy information about studies involving human research participants and Sex and Gender in Research.

Reporting on sex and gender	The subjects in this study self-identified as women.
Population characteristics	ONS data were grouped according to age, ethnicity, and index of multiple deprivation (IMD). Age was classified in three groups: 18-29, 30-39, and 40-49 years. Ethnicity was defined according to the set of standard codes used by the UK Government Home Office with 19 specific categories and their aggregated ethnic groups according to NHS records (Supplementary Table 1). The IMD also provided by ONS, uses postal code to give an overall measure of deprivation within a defined geographic area (known as a Lower-layer Super Output Area, roughly equivalent to a neighbourhood of 1000-3000 people), and incorporates the following domains: income, employment, educations skills and training, health deprivation and disability, crime, barriers to housing and services, and living environment.
Recruitment	These data were from national surveillance of COVID-19 vaccination in England, and are population-based.
Ethics oversight	Ethics approval was not required for this analysis, given use of aggregate, anonymised data already approved and in use for public health surveillance.

Note that full information on the approval of the study protocol must also be provided in the manuscript.

Field-specific reporting

Please select the one below that is the best fit for your research. If you are not sure, read the appropriate sections before making your selection.

☒ Life sciences ☐ Behavioural & social sciences ☐ Ecological, evolutionary & environmental sciences

For a reference copy of the document with all sections, see nature.com/documents/nr-reporting-summary-flat.pdf

Life sciences study design

All studies must disclose on these points even when the disclosure is negative.

Sample size	This was a convenience sample of all women of reproductive age in England, from 8 Dec 2020 to 15 Feb 2022.
Data exclusions	None.
Replication	Not applicable. We were interested in exploring associations with vaccine uptake and their independence. We did not build a model.
Randomization	Not applicable. This was not a randomised trial, but an analysis of surveillance data.
Blinding	Not applicable. This was an analysis of surveillance data.

Reporting for specific materials, systems and methods

We require information from authors about some types of materials, experimental systems and methods used in many studies. Here, indicate whether each material, system or method listed is relevant to your study. If you are not sure if a list item applies to your research, read the appropriate section before selecting a response.

Materials & experimental systems

n/a	Involved in the study	
☒	☐	Antibodies
☒	☐	Eukaryotic cell lines
☒	☐	Palaeontology and archaeology
☒	☐	Animals and other organisms
☐	☒	Clinical data
☒	☐	Dual use research of concern

Methods

n/a	Involved in the study	
☒	☐	ChIP-seq
☒	☐	Flow cytometry
☒	☐	MRI-based neuroimaging

Clinical data

Policy information about clinical studies

All manuscripts should comply with the ICMJE guidelines for publication of clinical research and a completed CONSORT checklist must be included with all submissions.

Clinical trial registration	Not applicable.
Study protocol	Not applicable.
Data collection	Not applicable.
Outcomes	Not applicable.
